# A Case of Total Retinal Detachment With Multiple Retinal Cysts Showing a Favorable Postoperative Course

**DOI:** 10.7759/cureus.82111

**Published:** 2025-04-11

**Authors:** Shota Mikuni, Yoshio Hirano, Takaaki Yuguchi, Hiroshi Morita, Tsutomu Yasukawa

**Affiliations:** 1 Department of Ophthalmology and Visual Science, Nagoya City University Graduate School of Medical Sciences, Nagoya, JPN

**Keywords:** oct (optical coherence tomography), optos ultra-widefield imaging, pars plana vitrectomy(ppv), retinal cyst, retinal detachment (rd)

## Abstract

A 29-year-old man was aware of vision loss in his left eye that had been present for about two years. At the time of his visit to our clinic, his best-corrected visual acuity (BCVA) was 20/16 in the right eye and 20/200 in the left eye. There were no abnormal findings in the anterior or intermediate translucent areas of either eye. The fundus examination revealed a retinal dialysis in the inferior nasal retina of the left eye, followed by multiple retinal cysts on the pole side, and total retinal detachment. The fundus of the right eye showed no abnormal findings of note. A diagnosis of rhegmatogenous retinal detachment (RRD) of the left eye was made, and cataract surgery, encircling, and vitrectomy were performed. Intraoperatively, retinal cysts that did not seem to affect closure of the tear were left untreated. Postoperatively, the fluid in the retinal cyst disappeared and retinal restoration was achieved. The BCVA recovered to 20/22. In this case, the retinal cyst may have formed as a result of prolonged retinal detachment. The results of this case suggest that surgical treatment of the retinal cyst and complete removal of the intraretinal fluid may not be necessary.

## Introduction

Retinal cysts usually occur in the outer retina, secondary to inflammation, vascular injury, trauma, or retinal detachment [1,2]. The incidence of retinal cysts is estimated to be 1-3% in eyes with rhegmatogenous retinal detachment (RRD) [2]. Although vitrectomy and scleral drainage have been attempted to remove subretinal and intraretinal fluid, surgical treatment of cysts that do not affect closure of the tear is usually considered unnecessary [3]. In this study, we report a case of RRD with multiple retinal cysts that was successfully treated.

## Case presentation

**Figure 1:**
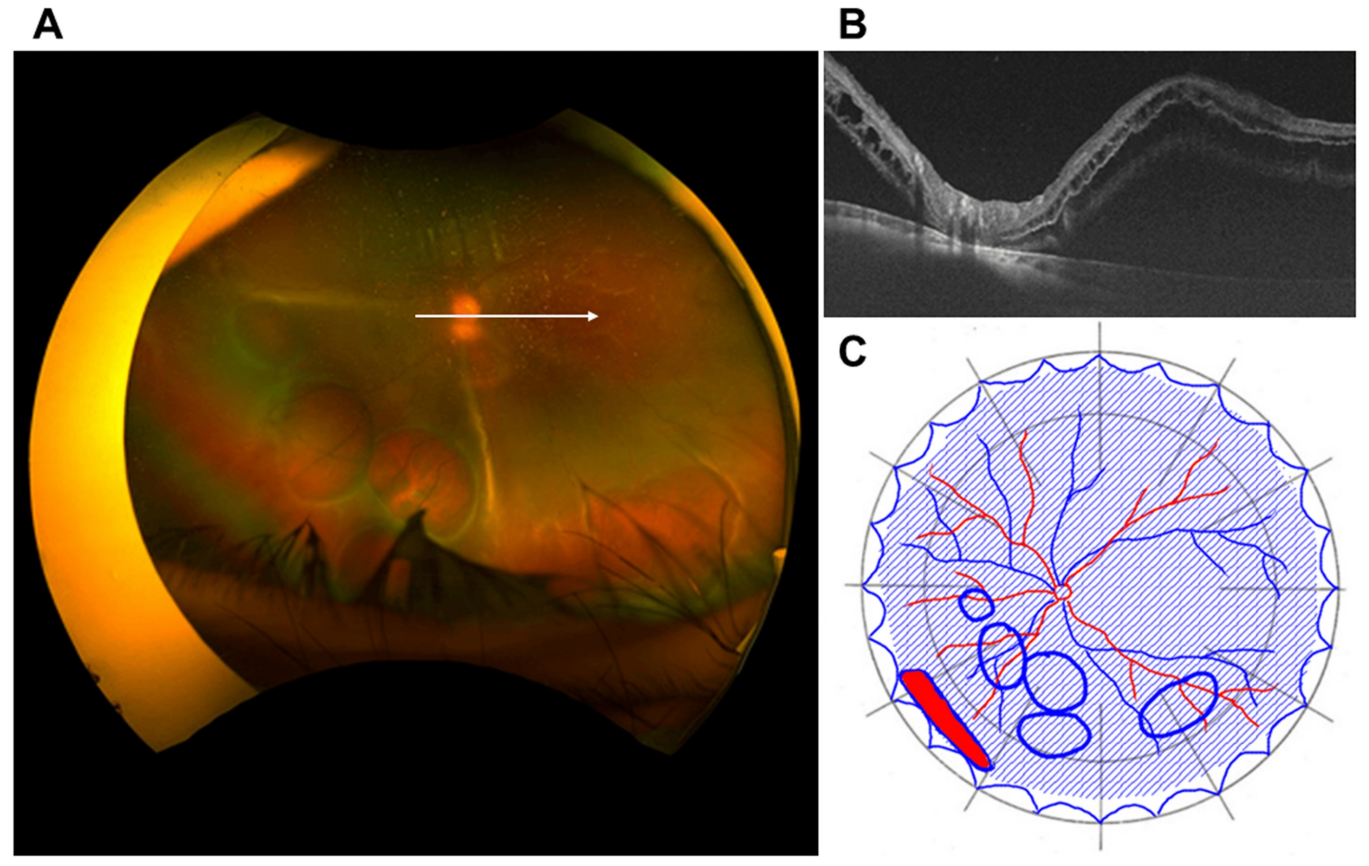
Preoperative findings A. Preoperative findings of ultra-widefield fundus photograph taken with Optos California (Nikon Corporation, Tokyo, Japan). Retinal dialysis was seen in the peripheral area of the inferior nasal side of the left eye, followed by multiple retinal cysts at the posterior side, and the retina was totally detached. B. Swept-source optical coherence tomography (SSOCT) findings of the white arrow in Panel A taken with Triton plus ver. 10.18. (Topcon, Tokyo, Japan). The retina was detached except for the optic disc, and the macula was also detached. C. Findings of fundus detachment chart. The blue shaded area indicates the detached retina, the red filled area with blue border indicates a retinal tear, and the blue circles indicate retinal cysts.

A 29-year-old man had noticed vision loss in his left eye for about two years. When he attempted to obtain a driver's license, he was advised to see an ophthalmologist due to vision loss. He was referred to our clinic because of RRD in his left eye after a visit to a nearby doctor. The BCVA of the left eye was 20/200. Intraocular pressure was 11 mmHg in the right eye and 13 mmHg in the left eye. Slit-lamp examination revealed no abnormalities in the anterior or intermediate translucent areas of either eye. Fundus examination revealed a retinal dialysis in the inferior nasal retina of the left eye, followed by multiple retinal cysts on the pole side, and the retina was in a state of total detachment (Figures 1A, 1B).

**Figure 2:**
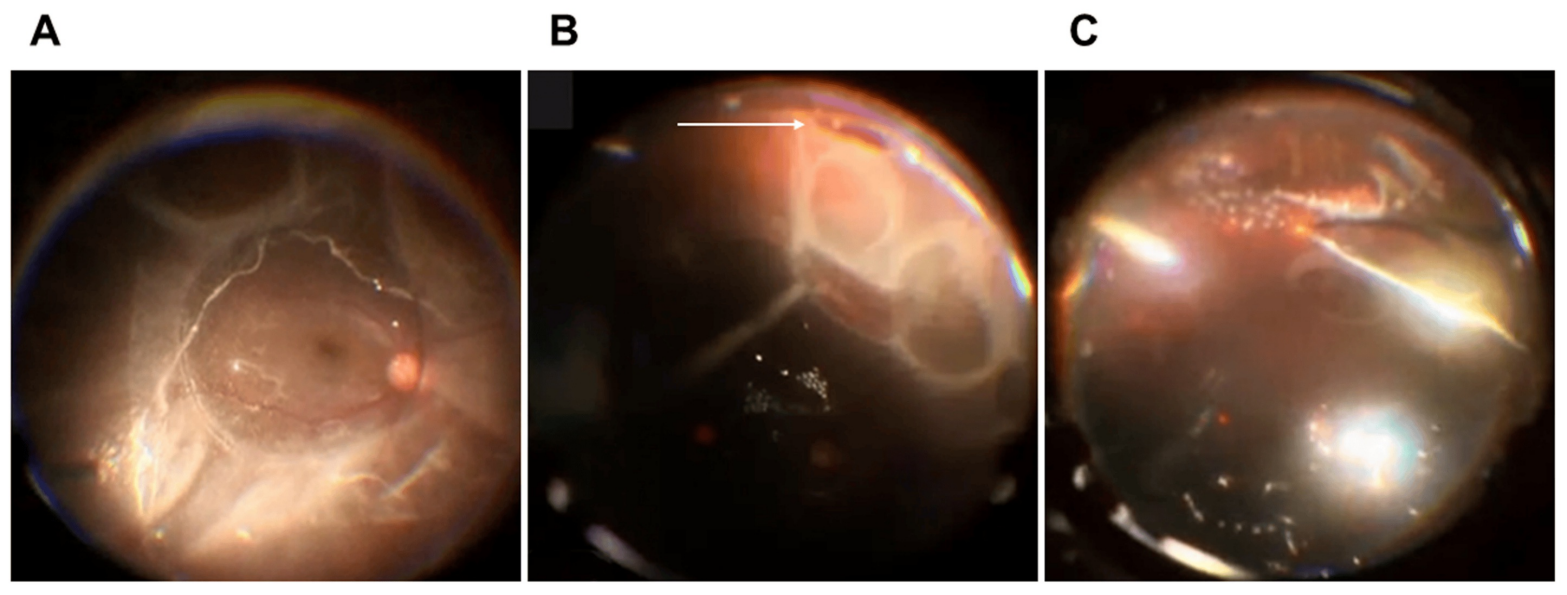
Intraoperative findings Intraoperative indirect fundus findings using Resight® 700 (Carl ZEISS AG, Jena, Germany). A. After creating a posterior vitreous detachment, perfluorocarbon liquid was injected into the posterior pole of the fundus to reattache the macula. B. A retinal dialysis (white arrow) was observed in the inferior nasal periphery of the retina, and a retinal cyst was present in contact with the posterior pole. C. After fluid-air exchange and retinal reattachment, endoretinal laser was applied around the retinal tear.

There were no abnormal findings in the fundus of the right eye. Optical coherence tomography (OCT) showed RRD extending into the macula of the left eye (Figure 1C). The left eye was diagnosed as rhegmatogenous retinal detachment with multiple retinal cysts, and cataract surgery, encircling, and vitrectomy of the left eye were performed approximately one month after the initial visit to our clinic. After creating a posterior vitreous detachment, perfluorocarbon (PFCL) was injected into the posterior pole of the fundus to restore the macula (Figure 2A).

**Figure 3:**
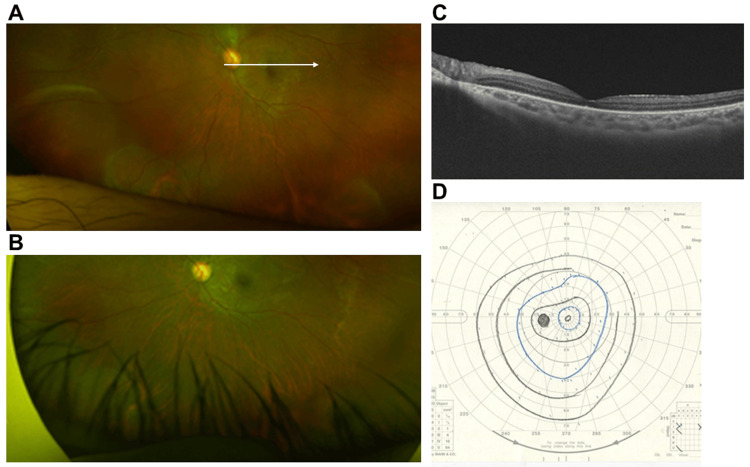
Postoperative findings A. Ultra-widefield fundus findings taken with Optos California (Nikon Corporation, Tokyo, Japan) one month after the surgery. The retina was almost reattached, but there were some residual fluids in the retinal cysts on the inferior nasal or inferior side. B. An ultra-widefield fundus photograph taken with Optos California one year after the surgery. The subretinal fluids were almost resolved and the retinal cysts were no longer evident. C. Swept-source optical coherence tomography (SSOCT) findings of the white arrow in Panel A taken with Triton plus ver. 10.18 (Topcon, Tokyo, Japan). The retina was almost reattached. D. Goldmannn perimetry findings of the left eye. Nasal and temporal visual field constrictions were present, but there was no visual field impairment corresponding to the area where the retinal cysts were located.

A retinal dialysis was observed in the inferior nasal periphery, which was thought to be the causative tear (Figure 2B). Further injections of PFCL drained the subretinal fluid from the causative tear, but some fluid remained in the retinal cyst. A thorough vitreous resection of the surrounding area was performed, and laser irradiation was performed around the tear (Figure 2C). Subsequently, liquid air displacement was performed, the PFCL was removed, and additional laser was applied. Sulfur hexafluoride (SF6) gas tamponade was performed. At the end of the surgery, some intraretinal fluid remained in the retinal cyst, although the fluid was not removed because it was thought not to affect closure of the tear, but the intraretinal fluid in the retinal cyst gradually disappeared after the surgery and the retina was restored (Figure 3A, 3B).

Postoperative Goldmann dynamic visual field testing showed nasal and temporal visual field narrowing in the left eye, but no visual field defects corresponding to the area of the retinal cyst were observed (Figure 3C). Postoperatively, the BCVA of the left eye recovered to 20/22.

## Discussion

A retinal cyst may be defined as a fluid-filled space in or derived from the retina, the diameter of which is greater than the thickness of the normal retina [1]. It is not necessarily a true cyst in the pathological sense, which must be lined by epithelium and have a discrete wall [1]. Retinoschisis is really the name of a process, not a condition, but it is commonly used when referring to the cysts which arise by this process [1]. Retinal cysts usually form in the outer retina, secondary to inflammation, vascular injury, trauma, or retinal detachment [1,2]. Their incidence is estimated to be 1-3% of RRD eyes [2]. Some retinal cysts have been reported to be associated with Coats' disease and toxoplasmosis and may be idiopathic [4-6]. It is sometimes necessary to differentiate retinal cysts from retinoschisis. In cases of retinoschisis, characteristic findings such as inner retinal layer dots on OCT and absolute scotomas on visual field testing, can help in making the distinction. In the present case, although OCT images of the lesion were not available, there were no absolute scotomas corresponding to the lesion on visual field testing. Based on this finding, the lesion was considered to be retinal cysts rather than retinoschisis. In this case, retinal cysts may have formed as a result of long-term retinal detachment. Pishel [7] classified retinal cysts into four groups as follows. 1) small cysts from microscopic size to 1 mm or so in size; 2) intermediate cysts large enough to be elevated several diopters with walls easily seen with the ophthalmoscope, perhaps four to eight disc diameters in size or even larger; 3) giant cysts, rising eight to 10 diopters or more into the fundus; 4) large cysts, occupying at least a quarter of the fundus. Basically, small cysts (1) can be treated with observation [7]. However, if the cyst is located in the upper half of the fundus and there is generalized posterior vitreous detachment, if a hole is seen in the cyst, or if RRD is seen in the symmetrical eye, prophylactic treatment is necessary [7]. Intermediate cysts (2) are often seen in patients who complain of visual disturbances and should be treated prophylactically because the cysts can cause retinal dialysis by traction on the retina [7]. Giant cysts (3) are often bilateral and are often temporally and inferiorly located. They often show activity such as cyst enlargement or the development of new cysts, and require surgical treatment [7]. Large cysts (4) are much less common than the other three categories [7]. They are thought to occur only in males, are often bilateral, and seem to be a recessive sex-linked trait [7]. The retinal cyst in this case was approximately 4 papillary diameters in diameter and was classified as an intermediate cyst (2), which requires prophylactic treatment [7]. The timing of surgery in such cases is often a matter of debate. Given the presence of macular detachment, early surgical intervention would generally be desirable. However, in the present case, the retinal detachment was considered long-standing, and we expected the visual prognosis to be limited. Consequently, the surgery was performed approximately one month after the initial visit. Fortunately, the visual outcome was better than anticipated. In this case, a retinal dialysis was observed, and it was necessary to ensure visibility of the retinal periphery in order to close the tear. Therefore, although the patient was young, he was treated with vitrectomy combined with cataract surgery. Surgical treatment of retinal detachment with retinal cysts has attempted to remove subretinal and intravitreal fluid by vitrectomy and scleral drainage [8], but treatment of retinal cysts is currently inconsistent. Cai et al. [9] concluded that vitrectomy with retinotomy was effective in removing giant cysts and relieving retinal traction. However, surgical treatment of cysts that do not affect closure of the tear is also considered unnecessary [3]. In a case report of seven cases of retinal detachment with retinal cysts by Marcus et al. [2], surgical treatment was not performed on cysts that did not affect closure of the tear, but in most cases the cysts disappeared after surgery. In the present case, some retinal cysts that did not affect closure of the tear remained during surgery, but the fluid in the retinal cyst disappeared after surgery, and retinal restoration was achieved. The BCVA recovered to 20/22, and there was no visual field defect corresponding to the portion of the retinal cyst. The results of this case indicate that surgical treatment of the retinal cyst and complete removal of the intraretinal cyst fluid may not be necessary. Chronic RRD is rare, with a prevalence of 4.5% to 29% of all RRD patients [10]. The initial retinal restoration rate for acute RRD is approximately 90% [11]. In contrast, the initial retinal restoration rate is only 57% for RRD that has been present for more than three months [12]. Among patients with chronic RRD, those without posterior vitreous detachment have a higher retinal restoration rate than those with posterior vitreous detachment [13-15]. In this case, the patient had been aware of vision loss for about two years and had a chronic RRD with macular detachment, but a good visual prognosis was achieved. The patient's young age was thought to be the reason for the good improvement in visual acuity.

## Conclusions

We report a patient with retinal detachment associated with multiple retinal cysts. This report may provide very useful information for both clinicians and patients regarding the treatment of retinal cysts. However, this is a case report. More detailed studies with a large number of cases are needed in the future.

## References

[1] Keith CG (1966). Retinal cysts and retinoschisis. Br J Ophthalmol.

[2] Marcus DF, Aaberg TM (1979). Intraretinal macrocysts in retinal detachment. Arch Ophthalmol.

[3] Brent AJ, El-Khayat AR, Peart SA, Banerjee S (2018). A case report of conservative management for a roller-coaster-related vitreous hemorrhage. Ophthalmol Ther.

[4] Karimi S, Nikkhah H, Fekri S (2019). Ocular toxoplasmosis presenting as subretinal macrocyst. J Ophthalmic Vis Res.

[5] Chen CY, Semenova E, Cohen BZ, Finger PT (2014). Idiopathic giant retinal cyst. Ophthalmic Surg Lasers Imaging Retina.

[6] Munira Y, Zunaina E, Azhany Y (2013). Resolution of exudative retinal detachment and regression of retinal macrocyst post-laser in Coats disease. Int Med Case Rep J.

[7] Pischel DK (1963). Surgical treatment of retinal cysts. Am J Ophthalmol.

[8] Wang Y, Cao X, Jia P (2024). Retinal giant cyst treated by the scleral buckling procedure: a case report. Medicine (Baltimore).

[9] Cai C, Zhou J, Wang Q, Li W, Liu D (2022). Case report: an intraretinal macrocyst with crystalline content and retinal detachment. Front Med (Lausanne).

[10] Sayman Muslubas I, Hocaoglu M, Ersoz MG, Arf S, Karacorlu M (2018). Choroidal thickness in chronic rhegmatogenous retinal detachment before and after surgery, and comparison with acute cases. Int Ophthalmol.

[11] Sahanne S, Tuuminen R, Haukka J, Loukovaara S (2017). A retrospective study comparing outcomes of primary rhegmatogenous retinal detachment repair by scleral buckling and pars plana vitrectomy in Finland. Clin Ophthalmol.

[12] James M, O'Doherty M, Beatty S (2007). The prognostic influence of chronicity of rhegmatogenous retinal detachment on anatomic success after reattachment surgery. Am J Ophthalmol.

[13] Li YM, Fang W, Jin XH, Li JK, Zhai J, Feng LG (2012). Risk factors related to chronic rhegmatogenous retinal detachment. Int J Ophthalmol.

[14] Yao Y, Jiang L, Wang ZJ, Zhang MN (2006). Scleral buckling procedures for longstanding or chronic rhegmatogenous retinal detachment with subretinal proliferation. Ophthalmology.

[15] Fang W, Li JK, Jin XH, Dai YM, Li YM (2016). Predictive factors for postoperative visual function of primary chronic rhegmatogenous retinal detachment after scleral buckling. Int J Ophthalmol.

